# Nanosystems in Cosmetic Products: A Brief Overview of Functional, Market, Regulatory and Safety Concerns

**DOI:** 10.3390/pharmaceutics13091408

**Published:** 2021-09-05

**Authors:** Chiara Ferraris, Clara Rimicci, Sara Garelli, Elena Ugazio, Luigi Battaglia

**Affiliations:** Dipartimento di Scienza e Tecnologia del Farmaco, Università degli Studi di Torino, Via Pietro Giuria 9, 10125 Turin, Italy; chiara.ferraris@unito.it (C.F.); clararimicci1996@gmail.com (C.R.); saragarelli.sg@gmail.com (S.G.)

**Keywords:** nanomaterials, cosmetics, dermal delivery

## Abstract

Nanosystems exhibit various innovative physico-chemical properties as well as a range of cosmetic functions, including increased skin retention for loaded compounds. The worldwide nano-market has therefore been consistently extensive in recent decades. This review summarizes the most important properties of nanosystems that are employed in cosmetics, including composition, functions and interactions with skin, with particular attention being paid to marketed products. Moreover, the worldwide regulatory landscape of nanomaterials used as cosmetic ingredients is considered, and the main safety concerns are indicated. In general, advanced physico-chemical characterization is preliminarily needed to assess the safety of nanomaterials for human health and the environment. However, there is currently a shortfall in global legislation as a universally accepted and unambiguous definition of a nanomaterial is still lacking. Therefore, each country follows its own regulations. Anyhow, the main safety concerns arise from the European context, which is the most restrictive. Accordingly, the poor dermal permeation of nanomaterials generally limits their potential toxic effects, which should be mainly ascribed to unwanted or accidental exposure routes.

## 1. Introduction

The development of innovative topical delivery systems is an exciting goal for researchers and the industrial field, especially because of the claimed advantages, such as the extensive cutaneous area and the easy handling of ready-to-use products. Nevertheless, the skin forms a barrier to substances applied to its external surface. Within this context, nanotechnology can be used to modify the process of permeation/penetration of bioactive substances through the skin by controlling their release and prolonging their residence time [1]. Moreover, it ensures direct contact can be made with the *stratum corneum* and skin appendages, while it also protects the loaded compounds against chemical and/or physical instability. Furthermore, the delivery of bioactive agents without the need for chemical enhancers is desirable as it can help to maintain normal skin-barrier function [1]. As a result, the use of nanocarriers for skin administration is expanding. 

Of the leading industrial sectors, the cosmetic sector was among the first to consider nanotechnology-based products, and it is currently a global leader in the incorporation of nanotechnologies in the development of new products [2]. In fact, a large number of nanosystems are commonly used in cosmetic formulations to encapsulate active ingredients [3]. The nanosystems employed in cosmetic products are made up of a variety of chemical compounds and compositions that are formulated in the nanometric range, and, owing to different supramolecular structures, include vesicular nanostructures (liposomes, niosomes), liquid nanoemulsions and nanoparticles [4,5]. The latter can be made up of matrixes of different origin and further divided into nanospheres (homogeneous matrices) and nanocapsules (core-shell structure). Moreover, when intended for cosmetic use, the final product itself can be formulated as a nanosystem (e.g., a cosmetic nanoemulsion), or, alternatively, a nanosystem can be included as a nano-ingredient in the final cosmetic product. The main targets of these innovative delivery systems for cosmetics include: improved skin retention for active ingredients, new colour elements (e.g., in lipsticks and nail polishes), transparency (e.g., in sunscreens), and long-lasting effects (e.g., in makeup). Indeed, their ultimate goal is to deliver the right amount of active ingredient to the desired parts of the body, and to attain long term stability [6]. However, although nanomaterials have aroused remarkable interest in the scientific community because of their atypical and innovative physico-chemical properties compared to bulk materials, there is distinct lack of an unambiguous and universally accepted definition of nanomaterial. The International Organization for Standardization (ISO) has defined “nanomaterial” as a “material with any external dimension in the nanoscale or having internal structure or surface structure in the nanoscale” [7], with nanoscale being defined as the size range from approximately 1–100 nm [8]. Nonetheless, these technical definitions, based on size only, may be insufficient from a risk evaluation standpoint, as they do not include other important elements that should be considered when determining whether a nanomaterial may need a more detailed assessment [9]. 

Indeed, cosmetic products for consumers are complex chemical matrixes. The nanosystem properties that can influence their interactions with the *stratum corneum*, as well as their potential deposition in furrows, appendages and deeper skin layers, include: size, shape, surface charge and properties (such as coatings or functional groups), and aggregation state. However, the vehicle in which they are suspended can have an influence as it can alter the substance’s properties and affect the permeability of the *stratum corneum* [5]. Therefore, the interactions between nanomaterials and other components of a formulation must be investigated to ensure the final cosmetic product’s performance and safety. Notably, with respect to safety, relevant concerns can be ascribed to the additional toxic effects that may arise from synergic mechanisms among ingredients that are employed as combinations and/or mixtures. Unfortunately, in some cases, similar unwanted effects cannot be adequately predicted despite the existence of toxicity data on individual ingredients.

Interestingly, the application of nanotechnology in skin care cosmetics can also promote the development of dermal (and transdermal) drug delivery. With the breakthroughs in preparation technology, quality control and mechanisms that promote permeability, it can be expected that the use of innovative nanosystems in the pharmaceutical field will increase in the near future [10]. Indeed, unlike in cosmetic products, in transdermal nanomedicines, transcutaneous permeation is a desirable phenomenon, which is governed by the same principles, and hampered by the same physiological barriers that act when a cosmetic product is applied to the skin. Therefore, the investigations that are ongoing into the mechanisms involved in the dermal permeation of nanosystems can have a relevant fall-out both in the cosmetics and the dermal/transdermal drug delivery fields. Within this concern, it should be noted that innovative nanosystems entered the cosmetic market first, and only afterwards arrived on the drug-delivery market. Generally, there is a typical time-frame between the invention of a nanosystem and its introduction onto the market. From the invention of liposomes in 1965, it took about 20 years for them to reach the cosmetics market (anti-aging product “Capture” launched by Dior in 1986) and 25 years for the first pharmaceutical product (Alveofact by Dr. Karl Thomae GmbH Germany) [11]. In fact, despite considerable effort by private pharmaceutical companies, the approval rate for novel nanomedicinal products by regulatory agencies does not exceeded 10%, mainly because of safety and efficacy-profile failures during clinical studies. Anyway, cosmetic products are easier to process in terms of time and economic investment, as they do not require clinical evaluation [12,13]. However, the current regulatory demands as to the safety of nanomaterials in the cosmetics field, as well as the relevant studies performed to assess this issue, could have implications on future pharmaceutical guidelines. To this aim, it is necessary to improve the development of harmonized regulations and establish a standardized evaluation system for the assessment of the efficacy and safety of cosmetic nanomaterials, that, in the future, might also be applied to nano-structured pharmaceutical preparations [10].

Within this context, the present review describes the most important properties of nanosystems that are used in cosmetics, such as composition, functions and interaction with the skin, and an overview of the nano-market is also included, both for cosmetic ingredients and finished cosmetic products. Moreover, the worldwide regulatory landscape of nanomaterials for use in cosmetic products is also considered, with the main safety concerns being highlighted. 

## 2. Formulation and Characterization of Nanosystems Used in Cosmetics

The most important nanosystems can be classified as [14]:

- 0D: all dimensions fall within the nanometer scale;
- 1D: with one non-nanoscale and two nanoscale dimensions;
- 2D: only one dimension in the nanometer range;
- 3D: materials with various dimensions below 100 nm, while combining multiple nanocrystals in different directions.

0D are usually employed in cosmetic products. They can be produced using “top-down” and “bottom-up” methods. In “top-down” techniques, also called destructive techniques, nanosystems are produced starting from materials of higher dimensions, so-called bulk materials, which are decomposed into smaller components. High energy is required to overcome the internal binding forces [15,16]. “Bottom-up” techniques, or constructive techniques, produce nanosystems through the assembly of relatively simpler atomic or molecular compounds [16]. This approach allows for better control of the desired characteristics, such as morphology and size, but it is limited by low yields. On the other hand, “top-down” methods are easier to reproduce on a large scale, but can lead to stability problems for the loaded actives, given the high temperatures and pressures required [15]. According to their chemical composition, nanosystems used in cosmetic products fall within the following categories: (1) lipid nanosystems (nanoemulsions, vesicles, lipid nanoparticles); (2) polymeric nanoparticles; (3) inorganic nanoparticles; and (4) carbon-based nanoparticles [15,17,18,19,20,21,22,23,24] (Table 1).

Despite the widespread use of nanosystems in cosmetic formulations, as well as the innovative properties of nanomaterials, relevant concerns regarding the safety of such nanomaterials as cosmetic ingredients are arising. Therefore, in order to determine whether a nanomaterial is safe for human health and the environment, a series of parameters should be evaluated [51,52]. To this aim, the suitable characterization of the nanomaterial should be performed. In the European Union (EU) this occurs according to the Scientific Committee on Consumer Safety (SCCS) “Guidance on the Safety Assessment of Nanomaterials in Cosmetics” [53]. The choice of key parameters to be measured and of the suitable characterization methods depends upon the composition, properties and foreseen use of the nanomaterial. This issue has been the subject of discussion in many international expert committees and working groups. In conclusion, the physico-chemical parameters that are considered relevant for the characterization of nanomaterials for safety assessment purposes have been reported in Table 2, together with the main analytical characterization techniques employed [15,24,51,52,53,54,55,56]. 

Both nanomaterial physical form and surface chemical reactivity should be considered: size, shape, morphology influence, deposit site, clearance and biological responses, while the interactions with organisms and the environment depends mainly upon the surface area and chemistry [51]. Indeed, risks to health and the environment may not only arise from the chemical composition of nanomaterials, but also from their size and surface features (e.g., coatings), which, in turn, can modulate absorption, biokinetics and toxic effects [53]. 

In this context, the determination of the chemical identity and composition of the nanomaterial, in terms of purity, presence of impurities, coatings, doping and encapsulated materials, processing chemicals, dispersing agents and additives, can be achieved using several spectroscopic techniques, according to the chemical nature of the material, which can be both organic (Mass Spectroscopy, Fourier Transformed Infra–RedFTIR, Nuclear Magnetic Resonance–NMR) and inorganic (Atomic Absorption Spectroscopy, Inductively Coupled Plasma-Mass Spectrometry, Mössbauer). Chromatographic techniques (High Performance Liquid Chromatography; Gas Chromatography) are also employed, with the aim of achieving the analytical separation of chemical moieties. Differential Scanning Calorimetry, together with X-Ray Diffraction, provides information about the crystalline state and potential polymorphisms. Particle size and morphology/structure determination are of primary importance: methods that are based upon light scattering (Dynamic & Static Light Scattering) and density/size separation (Field Flow Fractionation, Hydrodynamic Chromatography, Centrifugal Liquid Sedimentation) can be used to measure mean particle size and size distribution. The latter is very important in the case of agglomerates and aggregates, which are likely to have different chemical and biological properties than the isolated particles. However, the use of more than one characterization method is recommended for particle size, and can include electron microscopy-based imaging (Transmission Electron Microscopy & Scanning Electronic Microscopy), which are also of prior relevancy for the determination of shape (spherical, tube, rod), aspect ratio (for fiber/tube-like materials) and spatial distribution (e.g., homogeneous mixture, core-shell, surface coating). Surfaces should be characterized in terms of specific area (Brunauer Emmett and Teller) and characteristics. In particular, surface charge (Zeta potential) can be measured using light scattering (Laser Doppler Electrophoresis), reactive sites and surface functionalization/modification (i.e., coatings) can be detected using spectroscopic methods (FTIR, NMR, Raman Spectroscopy, X Ray Photoelectron Spectroscopy), and Gel Electrophoresis can be used in the case of protein ligands [15,24,51,52,54,55,56].

Moreover, further risks to health and the environment are posed if a nanomaterial loses its nanostructure. Therefore, the determination of nanomaterial stability, using the above-mentioned methods, is of primary importance. Indeed, since reactions that occur during handling or storage can modify interactions with biological systems, the characterization of nanomaterials in a cosmetic product should takes place in three phases: (1) at the manufacturing of the nanomaterial (pure state); (2) after the addition to the finished cosmetic (consumer exposure); (3) under usage conditions (toxicological investigation) [53]. 

## 3. Cosmetic Functions of Nanosystems

In the EU, a cosmetic product is defined as “any substance or preparation intended to be placed in contact with the various external parts of the human body (epidermis, hair system, nails, lips and external genital organs), or with the teeth and the mucous membranes of the oral cavity, with a view, exclusively or mainly, to cleaning them, perfuming them, changing their appearance and/or correcting body odours and/or protecting them or keeping them in good condition” [57]. A cosmetic product is therefore defined by its site of application (skin and annexes) and its primary functions, and excludes any therapeutic effects. Since intact skin is the primary application site for cosmetic products, many nanosystems claim to benefit the different layers of the epidermis (nourishment, hydration), or to penetrate further into the dermis to repair damaged cells and matrices (e.g., collagen, elastin), and these products include anti-aging ones [18,21,58]. However, according to the above definition, the skin penetration of cosmetic active ingredients, although desirable, must be strictly controlled to the viable epidermis and dermis, without reaching systemic circulation, as transcutaneous delivery exceeds the specific cosmetic functions [18]. 

Several different cosmetic functions can be attributed to nanosystems (Table 1), and these can be grossly divided into: (1) improvement of intrinsic properties of cosmetic ingredients and finished cosmetic products; (2) increased skin permeation (with the above-mentioned limitations). 

The former category includes: sustained release, increased physico-chemical stability, reduced irritability, improved textural quality and improved dispersion/spreading properties for the active cosmetic ingredients. Sustained release can be achieved via various mechanisms. Indeed, nanosystems can be divided into two major categories, according to their release properties: the first, which ‘disintegrates’ upon application onto the skin, includes vesicles and lipid nanosystems; the second, which remains ‘insoluble’ and ‘persistent’ throughout its usage, includes polymeric, inorganic and carbon based nanoparticles [21]. With regards to improvements in physico-chemical stability, nanosystems can be used for the protection of volatile compounds (such as perfumes), and sensitive compounds (such as hydroquinone) [59,60,61]. On the other hand, they can secure diminished direct skin contact with irritant molecules, such as dihydroxyacetone [59]. Finally, reducing the particle size to the nano range improves spreadability and provides transparency to titanium dioxide and zinc oxide, which are commonly used as physical UV filters [62].

**Figure 1:**
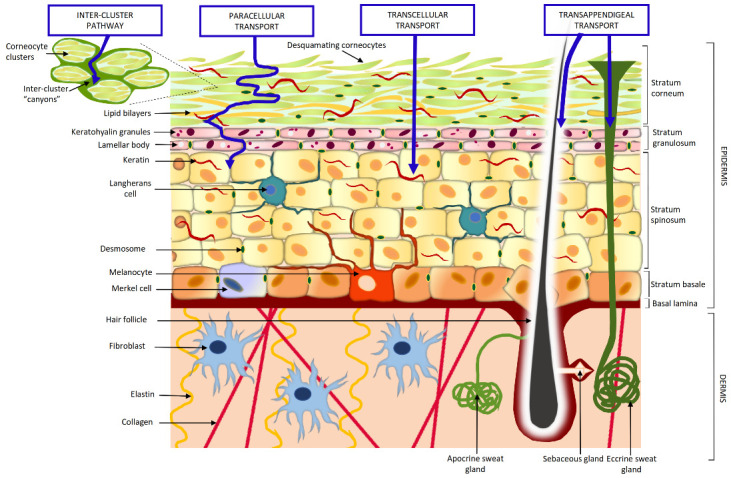
Absorption pathways through the skin.

The skin penetration of intact nanosystems, however, is a highly debated topic [63,64]. The suitable interpretation of literature data can come from a careful consideration of permeation pathways (transcellular vs. transappendageal) (Figure 1), size impact and the deepness of permeation [65]. The transappendageal route has no size limit (within the nanometer range), but is restricted to relatively small areas of the human body (less than 0.1% of the total surface), while the transcellular/paracellular ways are highly limited by the compact structure of the *stratum corneum*, which can be overcome only by lipophilic and low MW (<200 Da) molecules, owing to partition mechanisms [66]. Recent insights into skin structure have revealed that corneocytes in the *stratum corneum* are organized into clusters and surrounded by so-called “canyons”. These structures are filled with a non-polar, poorly hydrated material. With a size of nearly 20–30 nm, larger than the inter-corneocytes spaces (0.4 nm), they may allow the so-called “inter-cluster” pathway to be followed. However, it has been speculated that this permeation pathway may drive mainly in a lateral direction, while these “canyons” should act as a reservoir of hydrophobic compounds, due to their chemical composition [67,68,69,70,71].

**Figure 2:**
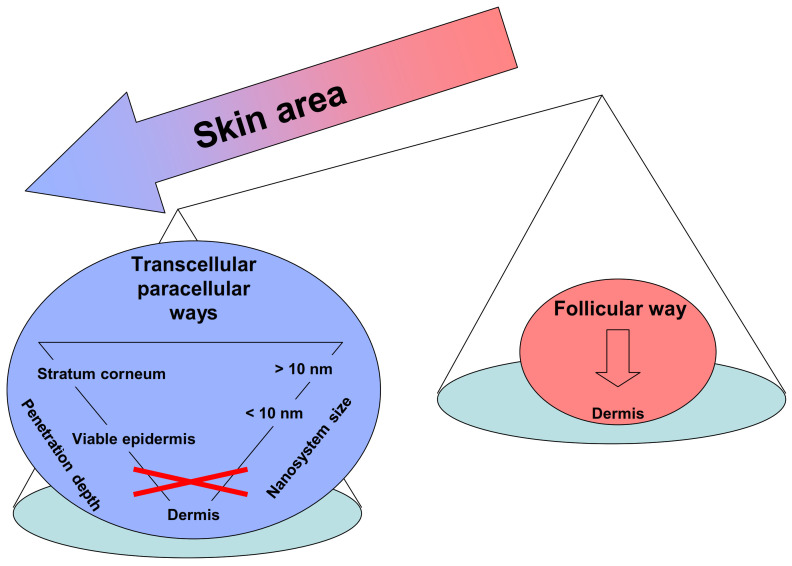
Permeation pathways of nanosystems through the skin.

This, in turn, reflects in the poor permeation of nanosystems through intact skin. An interesting article has reviewed the existing experimental work on the skin permeation of nanosystems, in terms of penetration path (transappendageal vs. transcellular/paracellular), depth and the influence of size [65]. Provided that diffusion via hair follicles and/or sweat glands contributes to skin permeation to a lesser extent, it was noticed that no nanosystem reached the dermis via the transcellular/paracellular route. Only a few nanosystems of those smaller than 10 nm showed some skin penetration, but this was limited to the viable epidermis, without reaching the dermis. Larger nanosystems were confined to the *stratum corneum* [65] (Figure 2). 

**Figure 3:**
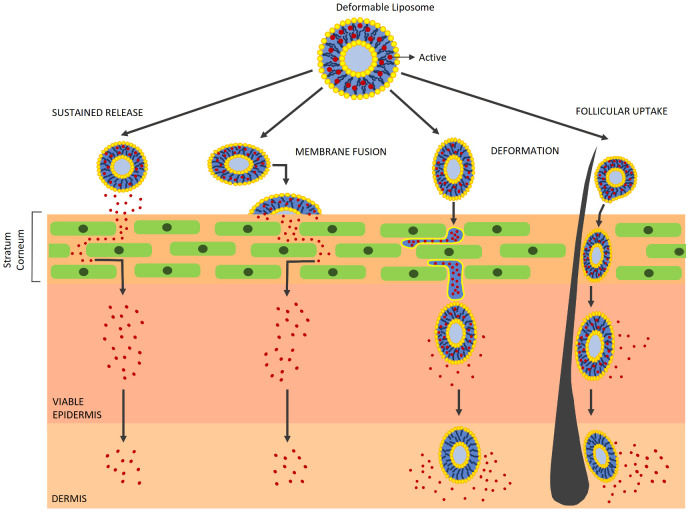
Mechanisms underlying the skin permeation of deformable liposomes.

A well-known exception to this rule can be found in deformable liposomes (i.e., transferosomes and ethosomes), in which surfactants and/or ethanol act as edge activators, allowing the vesicles to deform throughout the *stratum corneum* matrix. However, it is difficult to discriminate between their main claimed property and other simultaneous permeation mechanisms (Figure 3) [31,32]. IDEA AG holds the first international patents (1991 and later) that cover preparations based on such amphipathic aggregates, including those containing alcohols, which are mainly used for drug-delivery purposes [68]. Nonetheless, they can also be employed for cosmetic purposes [72]. Some marketed products are also based on ethosomes [73,74,75,76].

However, nanosystems can act as permeation enhancers for loaded active ingredients, via multiple indirect mechanisms, although intact nanosystems are unable to permeate. This is typical of lipid nanoparticles, which act as permeation enhancers via skin hydration and increased permeability, due to the occlusion effect of the lipids [25,26,27]. Moreover, surfactants that are used to formulate and/or stabilize such nanosystems play a key role in altering the compact structure of the *stratum corneum*, and therefore facilitate the skin permeation of compounds; this mechanism is particularly relevant for microemulsions, which are stabilized by highly concentrated surfactants [37].

## 4. Market Overview

The nano-market in cosmetic products began at the end of the eighties. The first liposome-containing marketed cosmetic product was the anti-age “Capture”, launched by Christian Dior in 1986 [77]. This was followed, in 1998, by “Plentitude Revitalift” (L’Oréal), an anti-aging cream containing polymeric nanocapsules for the delivery of active ingredients (e.g., retinol) [21]. This company allocated approximately 600 million $ (within its 17 billion $ profit) to “nano” patents [22,29], ranking them sixth among nanotechnology patent holders in the United States of America (USA) [22]. Pureology began employing nanoemulsions in 2000, when the company’s founder created a specific cosmetic line for hair dyes. In 2003, Caudalie, based in Paris, launched “Vinosun”, a sunscreen and anti-aging product containing nanometric UV filters and antioxidants [21]. In 2006, the cosmetics giant Estèe Lauder also began employing nanotechnology in its cosmetic products [22], followed by Procter & Gamble, Johnson & Johnson, Avon, Colorscience, and Doctor’s Dermatologic Formula [21,22]. Today, almost 40 years later, the use of nanotechnology in marketed cosmetic products worldwide is so widespread [18,19,20,21,22,78,79], that current attempts to categorize them (Table 3) fast become out of date, while updated distributions can be obtained from comprehensive market reports, performed by advanced technology consultancies [80]. 

On the other hand, additional information on marketed cosmetic products that contain nanosystems can be gathered from two freely available resources [80]. The first is the “Global Nanotechnology Database”, launched by StatNano in 2014 and regularly updated [87]. It includes three databases that cover international nanotechnology standards, global nanotechnology events and different countries’ policy documents. The second is the “Nanotechnology Consumer Products Inventory” (CPI) [88], a web resource created in 2005 by the “Project on Emerging Nanotechnologies”, together with the “Woodrow Wilson International Center for Scholars”, with the aim of gathering information on marketed “nano” products. To be included in the CPI, nanotechnology products must meet three criteria: (1) be easily purchased by consumers; (2) claim nanomaterial content, either by the manufacturer or by another source; (3) appear to be suitable in the opinion of the CPI curatorial staff [89]. However, this inventory is solely based on web information, and thus excludes any product that is not present on the internet. Furthermore, the CPI is updated by crowd-sourcing, thus allowing any user to suggest changes. This is a significant limitation as the CPI clearly specifies that neither the verification of the claims made by the manufacturers nor independent product tests are carried out [90]. According to these databases, cosmetic products represent 12% of the total nanomaterial-based products, while USA, Brazil, UK, Germany, France, South Korea, Russia, Poland, Switzerland and Malaysia are the top ten countries in promoting nanotechnology in the cosmetics industry. 

Nanosystems are employed in a wide range of marketed cosmetic categories: skincare and hair care, cleansers, make up and toothpastes [78,91]. Vesicles are mainly employed to improve skin retention and the release of anti-age, moisturizing, skin repair and whitening ingredients [81,82]. Besides liposomes, more recent nanosystems have also been marketed, and these include niosomes, the afore-mentioned ethosomes and novasomes, which are innovative multi-lamellar systems composed of synthetic surfactants [83]. Solid lipid nanoparticles (SLN), nanostructured lipid carriers (NLC) and nanoemulsions (NE) are made up of nourishing and moisturizing triglycerides and wax esters. Unlike SLN, which are exclusively composed of solid lipids, NLC contain mixtures of solid and liquid lipids, and provide an improved payload of the active ingredients [11]. From a technological point of view, the low energy Phase Inversion Temperature (PIT) technology, introduced by Vitacos Cosmetics to formulate an NE, is a relevant research innovation that is retained by marketed products. PIT technology couples small droplet sizes (<50 nm) and high lipid content (>20%), which, in turn, improves the retention of the loaded cosmetic ingredients in the deep skin layers [15,78]. Nonetheless, the comedogenic effect of the most commonly used lipids is a relevant hurdle. For instance, African Botanics was able to overcome this with an anti-age microemulsion based on a high concentration of natural and anti-comedogenic lipids, which are also suitable for acneic skins [37]. Polymeric nanoparticles are also widely used in marketed products, with the aim of improving the delivery of the loaded anti-age, nourishing and moisturizing active ingredients. Different proprietary nanotechnologies and/or “nano-brands” are retained by cosmetic companies, such as QuSome (Dr Brandt), and Liphazome (Dermazone Solutions) [22,81,84,85]. 

Moving now to inorganic nanoparticles, transparent nanopigments ZnO and TiO_2_, antimicrobial Ag, antioxidant Au and silica are the most reported [22,81,82,84,85]. Of particular interest from a technological viewpoint is Optisol, an innovative form of TiO_2_ that has been patented by Boots, and contains a small amount of manganese, which improves UVA protection and acts as a radical scavenger in sunscreen products [18,22]. Finally, recent market interest is growing towards fullerenes, which are carbon-based nanoparticles (C_60_), known for their strong antioxidant and antimicrobial activities, that are mainly used in anti-age and skin-repair products [21,22,79]. 

It should be noticed that certain nanosystems can only be used in some formulations, rather than all of them, depending on the cosmetic category and/or intended use. An additional reason for this is that, in some cases, there may be incompatibility between the nanosystem and the other cosmetic ingredients [92,93]; some metal oxides, such as zinc oxide, are very difficult to formulate at acidic pH [94]. Moreover, some nanosystems can alter the mechanisms of action of surfactants/emulsifiers and gelling agents, decreasing the stability of the final cosmetic products. Nano titanium dioxide is also photo-reactive with a resulting increase in reactive oxygen species (ROS), which are implicated in cellular damage. This issue has been solved by coating nanoparticles with alumina or silica to quench the production of ROS. This, in turn, also improves the dispersion of nanoparticles and their compatibility with other ingredients within sunscreen formulations [95]. Therefore, the engineering of innovative nano-ingredients that are suitable for use in different formulations is an emerging topic, and there are several examples of nanosystems that are marketed as cosmetic ingredients by chemical companies in order to be included in various finished products by cosmetics manufacturers (Table 4).

## 5. Regulatory Landscape

Currently, there is still a lack of an unambiguous definition-globally-that identifies nanomaterials as cosmetic ingredients. Therefore, each country follows its own definition and its own legislation [97]. As the EU and USA are the two largest markets for cosmetic products, a scheme of their regulatory frameworks is shown in Table 5.

### 5.1. EU

#### 5.1.1. Definition of Nanomaterial and Regulating Authorities

European Commission (EC) Regulation 1223/2009 provides a definition of nanomaterial for the cosmetic products, as “an insoluble or biopersistent and intentionally manufactured material, having one or more external dimensions, or an internal structure, on the scale from 1 to 100 nm” [57]. EC Recommendation 2011/696 updated this definition, in order to ensure compliance between different areas in which nanomaterials are used [99]. Accordingly, a nanomaterial is “a natural, incidental or manufactured material containing particles, in an unbound state or as an aggregate or as an agglomerate and where, for 50% or more of the particles in the number size distribution, one or more external dimensions is in the size range 1–100 nm. In specific cases and where warranted by concerns for the environment, health, safety or competitiveness the number size distribution threshold of 50% may be replaced by a threshold between 1 and 50%. By derogation from the above, fullerenes, graphene flakes and single wall carbon nanotubes with one or more external dimensions below 1 nm should be considered as nanomaterials” [99].

Regulation 1223/2009, released by the EC in 2009, governs cosmetic products in the EU. Moreover, the SCCS provides guidance for industries and public authorities to ensure compliance with Regulation 1223/2009, with particular concern to the safety assessment of ingredients that are intended for use in cosmetic products [6]. If the EC raises doubts about the safety of a nanomaterial, it can request an opinion from the SCCS, which formulates its evaluation within six months of the request. If the SCCS finds that there is a lack of necessary data, the EC asks the person responsible to provide such data within a reasonable period, which is explicitly indicated and cannot be extended. The opinion of the SCCS is then made publicly available [57].

#### 5.1.2. EC Released Documents

EC Regulation 1223/2009 provides specific rules for the labeling of cosmetic products that contain nanomaterials: each nanomaterial must be clearly indicated in the list of ingredients, placing the wording “nano” (in brackets) as a suffix to the name of the material. Furthermore, in the EU, all marketed cosmetics must name a responsible person (natural or legal), that has the task of supervising its conformity. Each cosmetic, before being marketed, must be electronically communicated to the EC through the Cosmetic Products Notification Portal (CPNP) by the responsible person, for market surveillance purposes. Additionally, the responsible person must communicate the existence of cosmetic products that contain new nanomaterials (that have not yet undergone full risk assessment by the SCCS) to the EC, in electronic format, six months before the products are marketed. Information provided should include nanomaterial identification, description (physico-chemical characterization), estimated amount marketed per year, toxicological profile, safety data (related to the cosmetic product) and exposure conditions [57].

#### 5.1.3. SCCS Released Documents

The SCCS, in 2021, issued the 11th revision of the “SCCS Notes of Guidance for the testing of cosmetic ingredients and their safety evaluation” [101], stating that the safety assessment of cosmetic products is based on the safety of the ingredients. This is established via risk assessment that is based on the level of exposure, which takes place on the basis of the toxicological data of the ingredients. To this aim, given the ban on animal testing for cosmetic ingredients in the EU [57], it is possible to consider the results reported in other relevant areas, but the use of this data must be duly supported and justified [101]. According to SCCS/1628/21, the safety evaluation of cosmetic products is based on the principles and practices of risk assessment that are usually applied for chemicals in the EU. Specifically, it is divided into four parts: (1) risk identification; (2) dose-response evaluation; (3) exposure assessment; (4) risk characterization [101]. Within these guidelines, reference is also made specifically to nanomaterials. For each cosmetic ingredient that meets the nanomaterial criteria, safety data is required from tests carried out, taking into account the properties of the nanoforms. 

Moreover, the SCCS, in 2019, published the document “Guidance on the Safety Assessment of Nanomaterials in Cosmetics” [53]. This Guide represents the revision of the 2012 Nanomaterial Safety Assessment document [104]. It was intended to take into account new developments in nanomaterial safety, and to facilitate applicants and risk assessors in the preparation of nanomaterial safety assessment dossiers [53]. It is mainly focused on risk identification (through physico-chemical characterization) and exposure assessment, as starting points for the safety assessment of nanomaterials. Recently, SCCS/1611/19 was updated in the “Scientific advice on the safety of nanomaterials in cosmetics” [105]. This document aims to identify those specific physico-chemical and exposure aspects, that constitute a concern for consumer safety, as well as to complete previously inconclusive safety assessments.

#### 5.1.4. Other Relevant Documents

In early 2020, the European Union Observatory for Nanomaterials (EUON) announced that all companies producing, using or importing nanoforms should be REACH (Registration, Evaluation, Authorization and Restriction of Chemicals) compliant registered. REACH is an EU Regulation issued in 2006 by the EC [106]. In December 2018, the EC updated Regulation 1907/2006 to include nanoforms [103].

### 5.2. USA

#### 5.2.1. Definition of Nanomaterial and Regulating Authorities

Unlike in the EU, in the USA, the Food and Drug Administration (FDA) has not yet approved a regulatory definition for nanomaterials and has stated that “*the current framework for safety assessment is sufficiently robust and flexible to be appropriate for a variety of materials, including nanomaterials*” [19]. However, scientists from the USA implicitly define nanomaterials as ranging between 1 and 100 nm [15,21], based on the definition given by some important organizations, such as the International American Society for Testing and Materials (ASTM), which is recognized worldwide for the development of international standards. In 2006, the ASTM published the first formalized definition of nanotechnology: “*any technology that measures, manipulates or incorporates materials and/or resources from 1 to 100 nm*”. This concept is very similar to the National Nanotechnology Initiative (NNI) definition: “*nanotechnology is the development, understanding and control of materials at the nanoscale, ranging from 1 to 100 nm*” [97]. 

In the USA, the FDA governs the use of nanotechnology in cosmetics [15] through the Federal Food, Drug, and Cosmetic Act (FFDCA) [98], which regulates a wide range of products besides cosmetics, including drugs and food [18]. The FDA has created the NNI and the Nanotechnology Task Force (NTF) to evaluate regulatory approaches for nanotechnology products [79]. In the USA, companies wishing to market cosmetics have a legal responsibility to ensure that their products and ingredients, including nanoscale materials, are safe and properly labeled [21]. Unlike the EU, in the USA, cosmetic ingredients do not need approval from regulatory agencies to be marketed, with the exception of dyes [15]. Moreover, the FDA does not require manufacturers to explicitly mention on the label that their products contain nanomaterials, since it is believed that the particle size is not necessarily related to the toxicity profile, and the labeling may therefore confuse consumers. However, the FDA has some regulations and procedures that cosmetic manufacturers can voluntarily choose to comply with. In fact, the FDA, together with the Personal Care Products Council (PCPC), has developed regulations on the voluntary registration of cosmetic ingredients and the reporting of adverse reactions. This occurs through the Voluntary Cosmetic Registration Program (VCRP). Through this program, manufacturers can be updated as to materials with known risks and, thus, remove them from their finished products. 

#### 5.2.2. FDA Released Documents

In June 2014, the FDA published three comprehensive guidance documents concerning the safety issues of nanotechnology: two of them are related to cosmetics. They do not establish legally taxable responsibilities, but only recommendations [18]. 

The first is “Considering whether an FDA-Regulated Product Involves the Application of Nanotechnology” [100]. Accordingly, two points are identified that should be used to assess whether FDA-regulated products, including cosmetics, involve the application of nanotechnology. They concern both the size of the particles and the properties/phenomena depending on size: (1) if “*a material or final product is designed to have at least one external dimension, or internal or surface structure, in the nanoscale range (approximately 1 nm to 100 nm)*”; (2) if “*a material or final product is designed to exhibit properties or phenomena, including physical or chemical properties or biological effects, which are attributable to its size, even if these dimensions are outside the nanoscale range, down to one micrometer (1000 nm*)”. The second point is very important because nanomaterial properties, which are relevant for safety, efficacy, performance, quality evaluation, public health impact and product regulatory status, can also be attributed to materials with one or more dimensions exceeding the 1–100 nm range [100]. 

The second document is the “Guidance for Industry-Safety of Nanomaterials in Cosmetic Products” [102]; safety should be assessed by characterizing the nanomaterial itself and evaluating a wide range of chemical and physical properties. The FDA stressed the importance of particle characterization in terms of: surface properties, morphological characteristics, other physical properties (i.e., solubility), agglomeration and dimensional distribution, possible presence of impurities [6,79]. Considerations as to the toxicology and absorption, distribution, metabolism and excretion (ADME) of nanomaterials in cosmetics can be obtained by considering the routes of exposure, uptake/absorption and toxicity tests. The exposure assessment for nanomaterials follows a procedure that is similar to the one for non-nano ingredients [6]. If necessary, traditional tests must be modified and new alternative methods may need to be developed as nanomaterial solubility can affect the suitability of a traditional method. Therefore, the FDA suggests adjusting traditional tests for insoluble or partially soluble nanomaterials, which is a rather common occurrence as nanoparticles frequently clump together, forming larger insoluble agglomerates [102].

### 5.3. Commonwealth Countries

Health Canada considers a nanomaterial “any substance or product manufactured and any component material, ingredient, device or structure if: (1) it is comprised within the nanometric dimensions in at least one external dimension, or has an internal dimension or surface structure within nanoscale, or (2) it is smaller or larger than the nanoscale in all dimensions, but exhibits one or more properties/phenomena of the nanoforms”. According to the Foods and Drugs Act, cosmetic products containing ingredients that are harmful to health should not be marketed. In 2007, Health Canada drew up a list of hazardous cosmetic ingredients, specifically a list of restricted or prohibited ingredients in cosmetics [79].

In Australia, the National Industry Chemicals Notification and Assessment Scheme (NICNAS) regulates the safety of ingredients in cosmetics and personal-care products, while sunscreens are regulated by the Therapeutic Goods Administration (TGA), and therefore considered as drugs. Neither of these associations, however, distinguishes between nanoparticles and bulk materials [22]. In Australia the definition of nanomaterial is provided by the NICNAS as: “*industrial material intentionally produced, manufactured or designed to have specific properties or a specific composition and one or more dimensions typically between 1 and 100 nm*”. As required by the TGA, all chemical ingredients, including natural ones, are regulated as industrial chemicals under the “Industrial Chemicals Notification and Assessment Act 1989” [79].

In New Zealand, the definition of nanomaterial is the same as in the EU. However, the adoption of a labeling system for nanomaterials that is similar to that in the EU, prior to international harmonization of the definition, would undermine the industry’s ability to standardize labels with other major markets, such as the USA and Australia, and thus inhibit trade and business opportunities. Therefore, according to the New Zealand authorities, the request for cosmetic labeling that explicitly refers to the presence of a nanomaterial should be temporarily set aside [79].

### 5.4. Other Countries

In Brazil, there are no regulations that are specific for nanomaterials and nanotechnologies. In 2012, ANVISA (National Agency for Sanitary Vigilance) promoted a debate on nanotechnology and security surveillance. In 2013, the Internal Committee of Nanotechology (CIN) was established with the aim of verifying the current understanding of nanomaterials. They prepared a document with the actions and regulatory policies on nanotechnologies present in other countries and suggested alternative guidelines and regulatory policies. [97].

In India, cosmetics are regulated by Schedule “S” of the Drugs and Cosmetics Act 1940 & Rules 1945, but there are no special provisions for assessing the safety or quality of cosmetics that contain nanomaterials [21]. The Bureau of Indian Standards (BIS) has established a special committee known as the “Nanotechnology Sectional Committee”, which is made up of 33 members from various research organizations and companies. This committee works for the standardization of nanotechnology regulations [79].

In China, cosmetic products are classified into two categories: ordinary and intended for a special use. Each of these requires a different type of license from the State Food and Drug Administration (SFDA). The latter have to undergo safety and health quality tests such as microbiological, toxicological, chronic toxicity, carcinogenic tests, and safe-for-human-use trials. For the marketing of cosmetics, a hygiene license or record-keeping certificate from the Health Administration Department of the State Council-SFDA must be obtained [78].

## 6. Safety Concerns

Although the unique properties of nanomaterials make them desirable as they can perform certain cosmetic functions, they may also represent a risk for consumer health. The concern that a potential health risk may be caused by insoluble nanoparticles is indeed a much debated topic in scientific literature, and this is mainly due to conflicting results and a lack of long-term toxicological studies [107,108,109,110,111]. The route of exposure plays a predominant role. The main route of exposure, as far as cosmetics are concerned, is cutaneous, but there are still uncertainties regarding the possibility that nanomaterials can penetrate through the *stratum corneum* and reach the vital layers. However, even if literature studies suggest generally limited skin absorption, this may increase in the case of damaged skin. Moreover, in safety assessment, special attention should also be paid to sprays or aerosols that contain nanomaterials, since exposure by inhalation is possible. Ingestion, on the other hand, can occur in the case of the use of cosmetic products that are applied to the mouth area (e.g., lipsticks), or through an involuntary transfer from hand to mouth [6].

The main evidence for the safety concerns about nanomaterials derives from the EU, since it has the most restrictive rules. Here, a nanomaterial refers to “any insoluble or biopersistent material”. This term could also refer to materials that are water-insoluble and biodegradable, especially with regards to those that consist of lipids (such as vesicles, SLN, NLC, etc.). However, these systems, due to their chemical nature and their similarity with physiological lipids present in the *stratum corneum*, are able to fuse with cell membranes, and are therefore considered soluble materials. The legislation specifically regulates those insoluble and biopersistent materials, for which there are greater toxicological concerns. Within this context, according to Regulation 1223/2009, by 11 January 2014, the EC made available a catalog of all nanomaterials used in cosmetic products, including those used as colorants, preservatives and UV filters in a separate section, indicating the categories of cosmetic products and reasonably foreseeable exposure conditions. Subsequently, this catalog is regularly updated and made publicly available [57]. The latest updated catalog provided by the EC consists of 29 nanomaterials and dates back to November 2019. This catalog is provided solely for consumer information, meaning that not all listed products are necessarily authorized [6], and is based on information that is electronically transmitted to the CPNP by the responsible person [15,112]. It should be noted that many of the listed substances are also registered under the REACH [6]. Of these, the EC has so far authorized the use of the following nanomaterials: UV filters containing nano-TiO_2_ [113,114,115], nano-ZnO [116], Methylene Bis-Benzotriazolyl Tetramethylbutylphenol (MBBT) [117,118] and Tris-Biphenyl Triazine [119]. The use of nano-Carbon black [120] as a colorant in cosmetic products is also allowed [6,15]. Accordingly, the Annexes of EC Regulation 1223/2009 have been updated, with the corresponding limitations of use. On the other hand, there are some nanomaterials (Nano-hydroxyapatite, Colloidal Silver, Silica, Styrene/Acrylate Copolymer, Colloidal Copper) for which the SCCS has expressed negative opinions, concluding that its use should be avoided in cosmetic products, or an inconclusive report due to a lack of data [121,122,123,124,125,126,127]. The most relevant safety concerns for nanomaterials employed in cosmetic products, for which a written opinion has been released by the SCCS, are summarized in Table 6.

## 7. Conclusions

Since the end of eighties, when nanosystems were introduced in cosmetic products, the worldwide nano-market has been consistently extensive due to the materials’ claimed advantages, including relevant cosmetic functions, such as the improvement of the intrinsic properties of the cosmetic product itself and increased skin retention of the loaded compounds. However, there is still no unambiguous definition-globally-that identifies nanomaterials as cosmetic ingredients, meaning that each country follows its own definition and legislation. Indeed, the main evidence for the safety concerns about nanomaterials in cosmetics comes from the EU, since it has the most restrictive regulations. Accordingly, in order to determine whether a nanomaterial is safe for human health and the environment, a series of parameters should be evaluated, and, in particular, advanced physico-chemical characterizations should be performed. However, it should be noted that the main safety concerns arise from accidental uptake through an unwanted exposure route, while the poor dermal permeation of insoluble and biopersistent nanomaterials limits their potential toxic effects. This aspect could also be important for dermal drug-delivery systems, given that, as previously mentioned, they have a longer time-to-market than cosmetics due to long-lasting clinical studies. Indeed, current disclosures as to poor absorption through the skin that are relevant for the safety assessment of cosmetic nanomaterials could pave the way, in the future, for a standardized safety assessment for nano-structured pharmaceutical formulations for skin applications.

## Figures and Tables

**Table 1:** Most commonly used nanosystems in cosmetics. Abbreviations: LUV: Large Unilamellar Vesicles; MLV: Multi-Lamellar Vesicles; NLC: Nanostructured Lipid Carriers; O/W: Oil in Water; SLN: solid lipid nanoparticles; SUV: Small Unilamellar Vesicles; W/O: Water in Oil.

Category	Type	Subtype	Features	Claimed Advantages	References
Lipid	Lipid nanoparticles	SLN, NLC	Solid (SLN) or solid/liquid (NLC) matrix-based nanoparticles stabilized with surfactants	High compound payload, stability, skin occlusion	[25,26,27]
	Vesicles	Liposomes (SUV, LUV, MLV)	Phospholipid-based vesicles	Water-soluble and lipid soluble compound loading in inner core and bilayer, respectively	[28,29,30]
		Elastic liposomes	Deformable liposomes, due to edge activator surfactants (transferosomes) or ethanol (ethosomes)	Overcoming the *stratum corneum*	[31,32]
		Niosomes	Synthetic surfactant (sorbitans)-based vesicles	Cheaper and more stable than liposomes; skin permeation enhancers due to surfactants	[28,33]
	Nanoemulsions	O/W; W/O	Submicron sized emulsion	Prolonged release of loaded compounds, skin occlusion	[34,35,36]
	Microemulsions	O/W; W/O	Clear ternary systems (oil, surfactant + co-surfactant, water)	Skin permeation enhancers due to high content of surfactants	[37]
	Cubosomes		Amphiphilic lipid based 3D honeycomb-like structures	Cheap, stable, prolonged release of loaded compounds	[38]
Polymeric	Nanospheres		Uniform matrix nanoparticles	Bioadhesion, prolonged release of loaded compounds	[39,40]
	Nanocapsules		Core-shell nanoparticles	Bioadhesion, prolonged release of loaded compounds	
	Nanofibers			Cheap, prolonged release of loaded compounds	
Inorganic	Metal	Ag, Au		Antimicrobial (Ag), Antioxidant (Au), Nanopigments	[41,42]
	Metal Oxides	ZnO, TiO_2_		Transparent physical sunscreen	[19,43]
	Silica (SiO_2_)			Prolonged release of loaded compounds	[44,45]
	Hydroxyapatite			Teeth remineralization in oral care products	[46,47]
Carbon-based	Fullerenes		C_70_, C_76_, C_84_, C_90_ e C_36_ but mainly C_60_ buckyballs	Antioxidants, antimicrobial	[48]
	Carbon nanotubes			High compound payload and prolonged release	[49]
	Other		Carbon dots, graphene and nanodiamonds, etc.	High compound payload and prolonged release	[50]

**Table 2:** Most important characterization methods for nanomaterials. Abbreviations: AAS: Atomic Absorption Spectroscopy; AFM: Atomic Force Microscopy; BET: Brunauer Emmett and Teller; CLS: Centrifugal Liquid Sedimentation; DSC: Differential Scanning Calorimetry; DLS: Dynamic Light Scattering; FFF: Field Flow Fractionation; FTIR: Fourier Transformed Infra-Red; GC/LC-MS: Gas Chromatography/Liquid Chromatography-Mass Spectroscopy; GE: Gel electrophoresis; HDC: Hydrodynamic Chromatography; HPLC: High Performance Liquid Chromatography; ICP-MS: Inductively Coupled Plasma-Mass Spectrometry; IR: Infra-Red; LDE: Laser Doppler Electrophoresis; MS: Mass Spectroscopy; MW: Molecular Weight; NM: nanomaterial; NMR: Nuclear Magnetic Resonance; RS: Raman Spectroscopy; SEM: Scanning Electronic Microscopy; SLS: Static Light Scattering; TEM: Transmission Electronic Microscopy; UV–Vis: Ultraviolet-Visible; XRD: X Ray Diffraction; XPS: X Ray Photoelectron Spectroscopy.

Information Category	Relevant Parameters for NM Characterization	Analytical Technique	Specific Information Gathered
Chemical identity	formula/molecular structure of the NM constituents	AAS	metal/inorganic content
		FTIR	functional groups: chemical structure
		ICP-MS	metal/inorganic content
		Mössbauer	metal/inorganic content
		MS	molecular ion, fragmentation spectrum: MW, chemical structure
		NMR (^1^H and ^13^C)	functional groups: chemical structure
Chemical composition	purity; nature of impurities; coatings/surface moieties; doping material; encapsulated materials; processing chemicals; dispersing agents; other additives (i.e., stabilizers)	AAS	metal/inorganic impurities & doping materials
		DSC	calorimetric transitions: melting temperature/enthalpy; polymorphism
		FTIR	functional groups: doping materials, processing chemicals
		GC/LC-MS	analytical separation & identification: purity; nature of impurities, encapsulated actives, processing chemicals
		HPLC	analytical separation: purity, encapsulated actives, processing chemicals
		ICP-MS	metal/inorganic impurities & doping materials
		Mössbauer	metal/inorganic impurities & doping materials
		NMR (^1^H and ^13^C)	funtional groups: doping materials, processing chemicals
		SEM	elemental analysis
		UV–Vis	functional groups, UV extintion coefficient: chemical structure
Crystallographic structure	crystalline form: amorphous, polycrystalline, crystalline; phase/volume fraction; spatial distribution	DSC	calorimetric transitions: liquid crystals; polymorphism
		XRD	crystal structure
		TEM	2D transmitted electronic image
Particle size and size distribution	distribution diagrams for agglomerates/aggregates: number versus size; number weighted sum function -cumulative numbers; batch-to-batch variation	AFM	probe scan image
		CLS	density/size separation
		DLS	mean particle size and polydispersity, size distribution
		FFF/HDC	size/MW based separation: size distribution, presence of agglomeration or aggregation
		SEM	3D backscattered electronic image
		SLS	mean particle size
		TEM	2D transmitted electronic image
Morphology/Shape/Structure	state/physical form: powder, solution, suspension; shape: spherical, tube, rod; aggregation: primary particulates/agglomerates; spatial distribution: homogeneous mixture, core-shell, surface coating	AFM	probe scan image
		SEM	3D backscattered electronic image
		TEM	2D transmitted electronic image
		XRD	crystal structure
Surface characteristics	surface charge: Zeta potential; morphology/topography; interfacial tension; reactive sites; chemical/biochemical modifications/coatings; surface contaminants	FTIR	functional groups: reactive sites, coatings, surface moieties
		GE	MW based separation: coatings/functionalization with proteins
		LDE	Zeta potential
		NMR (^1^H and ^13^C)	functional groups: reactive sites, coatings or surface moieties
		RS	surface binding, coatings, surface moieties of Carbon based materials
		XPS	surface elemental analysis
Surface area	specific surface area. volume-specific surface area	BET	surface area calculation by gas absorption
Concentration	particle mass/ particle number per volume	AAS	metal/inorganic dose quantification
		GC/LC-MS	analytical separation: dose quantification
		HPLC	analytical separation: dose quantification
		ICP-MS	metal/inorganic dose quantification
		UV–Vis	dose quantification
Stability	stability/dissociation constants in relevant formulation/media	DLS	mean particle size and polydispersity; size distribution
		DSC	calorimetric transitions: polymorphism
		FTIR	functional groups: chemical structure
		GC/LC-MS	analytical separation & identification: dose quantification
		HPLC	analytical separation: dose quantification
		MS	molecular ion, fragmentation spectrum: chemical structure
		NMR (^1^H and ^13^C)	functional groups: chemical structure
		SLS	mean particle size

**Table 3:** Most known examples of marketed cosmetic products that employ nanosystems. Abbreviations: HA: hydroxyapatite; ME: microemulsions; NC: nanocapsules; NE: nanoemulsions; NLC: nanoxtructured lipid carriers; NP: nanoparticles; NS: nanospheres; Nsom: nanosomes; SLN: solid lipid nanoparticles.

Type	Product Category	Nanosystem Function	Company *Line* & Product Name(s)	Ref.
Vesicles				
Liposomes	Hair care	Hair repair	**Sesderma** Seskavel Mulberry Anti-Hair Loss Foam	[10]
	Skin Cleanser	Skin purification	**Dermaviduals** Acnel Lotion N	[18]
	Skin care	Anti-age	**Aubrey Organics** Lumescence Eye Cream	[78]
			**Christian Dior** Capture Totale	[78,81,82]
			**I-Wen Naturals** Ageless Facelift Cream	[82]
			**Jafra Cosmetics** Royal Jelly Lift Concentrate	[82]
			**Kaya Skin Clinic** Derma Stemness Reviving Serum	[10]
			**Lucas Meyer** Isocell MAP	[10]
		Anti-age & moisturizing	**Russell Organics** Liposome Concentrate	[78]
			**Sesderma** C-Vit Liposomal Serum; Fillderma Lips Volumizer; Acglicolic Classic Crema Hidratante SPF 15; Daeses Lifting Cream	[10,15,78]
		Anti-age & skin repair	**Clinicians Complex** Liposome Face and Neck Lotion	[78,81]
			**Rovi Cosmetics Int** Rovisome ACE Plus	[82]
		Moisturizing	**Dead Sea Premier** Bio Performance Liposome	[10]
			**Decorte** *Moisture Liposome*: Eye Cream/Face Cream	[78,81]
			**Kerstin Florian** Rehydrating Liposome Day Creme	[78,81]
			**Microfluidics** Dermosome	[78,81]
			**Nattermann PL** Natipide II	[20]
		Skin repair	**Estee Lauder** Advanced Night Repair Protective Recovery Complex	[78,81]
		Whitening	**Sesderma** Azelac Ru Serum	[10]
Niosomes	Hair Cleanser	Hair repair	**Identik** *Floral Repair*: Shampooing	[78]
	Hair care		**Identik** *Floral Repair*: Masque	[78]
	Make up	Anti-age & skin repair	**Lancome** Niosome + Clear whitening foundation cream	[78,81]
	Skin care	Anti-age & skin repair	**Eusu** Niosome Makam Pom Whitening Facial Cream	[78,81]
			**Lancome** Niosome + Perfected Age Treatment	[78,81]
			**Laon** Cosmetics Mayu Niosome Base Cream	[78,81]
			**Simply Man Match** Anti-Age Response Cream	[78,81]
		Whitening	**Guinot** Deep action lightening serum	[10]
Ethosomes	Skin care	Anti-age & skin repair	**Genome Cosmetics** Decorin Cream	[74,75,76,77]
		Adjuvant for cellulitis	**Hampden Health** Cellulight EF	[74,75,76,77]
			**NovelTherpeutic Technologies** Noicellex	[73,75,76,77]
			**Osmotics** Lipoduction	[73,77]
			**Physonics** Skin genuity	[76,77]
Novasomes	Skin care	Moisturizing	**Amore Pacific** *Water Bank*	[83]
			**IGI** *MIAJ*	[83]
			**Jo. & Johnson** *Neutrogena*	[22]
NSom	Skin care	Anti-age	**L’Oreal** *Revitalift*: double lifting/intense lift treatment mask	[15,18,22,82,84]
Lipid NP				
SLN	Parfum	Stabilizer	**Chanel** *Allure*: Eau Parfum Spray/Parfum Bottle	[78,81]
	Skin care	Anti-age & skin repair	**Soosion** Facial Lifting Cream SLN technology	[81]
		Moisturizing	**Chanel** *Allure*: Body Cream	[78,81]
			**Yamanouchi** Nanobase	[11]
NLC	Skin care	Anti-age	**Scholl** Regenerations Cream Intensive Ampoules	[11,78]
		Anti-age & skin repair	**Amore Pacific** *Iope Supervital Extra Moist*: Eye Cream	[11,78]
			**Beate Johnen** *NLC deep effect*: eye serum/repair cream/reconstruction cream	[11]
			**Chemisches Laboratorium (Dr. Richter)** *NanoLipid:* Basic/Q10/Repair/Restore	[11]
			**Dr. Rimpler** *Cutanova Cream*: Nano Repair Q10/ NanoVital Q10; Intensive Serum NanoRepair Q10	[11,78,82]
			**Dr.Theiss (Medipharma Cosmetics)** *Olivenol*: Anti Falten Pflegekontrat/Augenpflegebalsam	[11,78]
			**Sirech Emas** Phyto NLC Active Cell Repair	[15,81]
		Moisturizing & skin repair	**Amore Pacific** *Iope Supervital Extra Moist*: Softener	[11,78]
			**Isabelle Lancray** *Surmer*: Crème Contour Des Yeux Nano-Remodelante/Creme Legere NanoProtection/Crème Riche Nano-Restructurante/Elixir du beauté Nano-Vitalisant/Masque Creme Nano-Hydratant	[11]
		Skin repair	**La Prairie** Swiss Cellular White Illuminating Eye Essence	[11,78]
NE & ME				
NE	Hair care	Anti-fading	**Korres** Red Vine Hair Sun Protection	[78]
			**Pureology** Color Max	[22]
	Parfum	Stabilizer	**Chanel** Calming Alcohol Free Nanoemulsion	[22,78,81,84]
	Skin care	Anti-age	**La Prairie** Skin Caviar ampoules	[15,22,84]
			**MiBelle Biochemistry** *Nano-LipoBelle*: H-AECL/E-Q10 Cream	[82]
			**Marie Louise** Vital Nanoemulsions A-VC	[81]
		Anti-age & moisturizing	**Bayer HealthCare** Bepanthenol-Protect Facial Cream Ultra	[78]
			**Rhonda Allison** Phyto-Endorphin Hand Cream	[78,81]
		Moisturizing	**Chanel** Precision-Solution Destressante Solution NanoEmulsion Peaux Sensitivity; Coco Mademoiselle Fresh Moisture Mist	[22,78,81,84]
			**Coni Beauty** Hyaluronic Acid & Naneomulsion Intensive Hydration Toner	[78,81]
			**Vitacos Cosmetics** Vita-Herb Nona-Vital Skin Toner; Nanovital Vitanics Crystal Moisture Cream	[15,78,81]
ME	Skin care	Anti-age	**Auriga International** Aurigene Microemulsion P	[37]
		Skin repair	**African Botanics** Cloudburst Microemulsion Balancing Moisturizer	[37]
Polymeric NP				
NS	Skin Cleanser	Skin purification	**Kara Vita** Clear It! Complex Mist	[18,78,81,85]
	Hair care & skin care	Nourishing	**Pureology** *Nanowax*	[18]
	Skin care	Anti-age	**Cell Act Switzerland** DNA Filler Intense Cream	[81]
			**Dermaswiss** Nanosphere Plus	[81,82]
			**Kara Vita** Eye Tender; Lip Tender	[18,78,81,85]
		Moisturizing	**Coryse Salome Paris** Competence Hydration Ultra-Moisturizing Cream	[78]
			**Hydralane Paris** Ultra Moisturizing Day Cream	[78,81]
			**Kara Vita** Fresh As A Daisy Body Lotion	[18,78,81,85]
		Skin repair	**Dermazone Solutions (Lyphazome)** Moisturizing sunscreen MAX SPF 29/Moisturizing sunscreen SPF 30	[15,18,22]
		Whitening	**Kara Vita** Enlighten me	[18,78,81,85]
NC	Skin care	Anti-age	**Dr. Brandt (QuSome)** Double dose in a box; Laser relief; Laser tight	[22]
			**Eccos** Nano vita C	[15]
			**Euoko** eye contour nanolift	[10]
			**Pharmanex** LifePak Nano	[85]
		Anti-age & skin repair	**Lancome** Hydra flash bronzer; Soleil Instant Cooling Sun Spritz SPF 15; Primordiale Optimum Lip; Hydra Zen Cream	[15,22,82,84,85]
		Moisturizing	**Enprani** Super aqua skin cream line	[22,84]
Inorganic NP				
Au	Skin care	Anti-age	**Orogold** 24 K Nano Ultra Silk Serum	[78,81]
			**Chantecaille** *Nano Gold Energizing*: Cream/Eye Serum	[78]
			**Nuvoderm** Nano Gold Anti-Aging Lifting Serum	[78]
			**LR Zeitgard** Nano Gold & Silk Day Cream	[78,81]
			**Lexon** Nanorama—Nano Gold Mask Pack	[85]
			**Ameizii** Nano Gold Foil Liquid	[78,81]
		Anti-age, whitening	**Tony Moly** Nano Gold BB Cream SPF 50 PA+++	[78]
		Whitening	O3+ 24 K Gold Gel Cream	[78,81]
Ag	Skin Cleanser	Skin purification	**NanoCyclic** Cleanser Silver	[85]
			**Natural Korea** *Cosil*: Nano Beauty Soap	[85]
	Skin care		**Natural Korea** *Cosil*: Whitening Mask	[85]
Au + Ag	Skin care	Anti-age & skin purification	**Joyona International Marketing** Nano Gold 24 Hour Cream	[18,21,79]
ZnO	Skin Cleanser	Adsorbent	**Nano-Infinity Nanotech** Nano-in Deep Cleaning	[18,21]
	Skin care	Sunscreen	**Antaria** Zinclear	[84]
			**Dermatone** Moisfurizing lips ‘n face protection crème	[22]
			**Procter & Gamble** Olay complete UV protective moisture lotion	[22]
TiO_2_	Skin care	Sunscreen	**Boots (Optisol)** Soltan facial sun defence cream	[22]
			**Christian Dior** DiorSnow Pure UV Base SPF 50	[82,85]
ZnO + TiO_2_	Make up	Sunscreen	**Colore Science** *Sunforgettable*: corrector colores SPF 20/SPF 30 brush range	[22,84]
	Skin care		**Colore Science** Wild to mild skin bronzer	[22,84]
SiO_2_	Skin care	Antiage & skin care	**Global Med Tech.** Leorex hypoallergenic wrinkle nano remover line	[22,84]
			**Lancome** *Renergie*: lift makeup/microlift eye	[22,82,84]
			**Shiseido** Elixir skin range; Pureness matifying compact	[22]
Al_2_O_3_	Make up	Absorbent & anti-caking	**Revlon** Colorstay natural powder; New complexion concealer	[22]
Mica	Hair care	Colorant	**Pureology** Nano Works Shine Luxe	[18]
	Make up		**Colore Science** Dual Finished Pressed Compacts	[18]
HA	Toothpaste	Abrasive	**Apagard** Apagard Premio toothpaste	[86]
Carbon based				
Fullerenes	Skin care	Anti-age	**Dr. Brandt** New lineless cream	[22]
			**Sircuit Cosmeceuticals** White out/Daily under eye care	[21,22,79,82]
		Anti-age & skin repair	**Bellapelle Skin Studio** *Defy*: Age management exfoliator/EGF complex cocktail/Nourish	[18,22]
			**MyChelle Dermaceuticals** Revitalizing night cream	[22]
		Skin repair	**Zelens** *Fullerene C_60_*: day cream/night cream	[22,82]

**Table 4:** Most known examples of nanosystems marketed as cosmetic ingredients. Abbreviations: DHA: dihydroxyacetone; O/W: oil in water.

Company	Nanosystem	Product(s) Name	Function	Ref.
	Vesicles			
Applied genetics	Enzyme loaded	Ultrasomes; Photosome	Suncare products, skin repair	[73,96]
BASF	DHA loaded	Elespher	DHA protection & delivery	[96]
BASF		Catezome	Improved skin delivery	[96]
	Nanoemulsions			
Sinerga	O/W	Nanocream	Sprayable, hyperfluid emulsions; wet wipes	[78]
	Microemulsions			
Abitec	Oily	Caprol Microexpress blends	Sunscreens & anti-age actives delivery	[37]
Dow Corning	Silicon	Dowsil	Hair care: conditioners, gels	[37]
	Nanoparticles			
Advanced Nanotechnology	Al_2_O_3_	Alusion Aluminum Powders	Adsorbent & anti-caking	[18]
Advancec Polymers Systems	Polymer	Microsponge	Improved skin delivery	[59,96]
BASF	Nylon/silica	Elesponge	Emollient	[96]
Degussa	TiO_2_	Tego Sun TS Plus	Sunscreen	[82]
Micronisers Pty	ZnO	NanoSun	Sunscreen, adsorbent	[82]

**Table 5:** Scheme of the regulatory frameworks for nanomaterials in cosmetic products in EU and USA. Abbreviations: ASTM: American Society for Testing and Materials; EC: European Commission; EUON: European Union Observatory for Nanomaterials; FDA: Food and Drug Administration; NNI: National Nanotechnology Initiative; NTF: Nanotechnology Task Force; PCPC: Personal Care Products Council; REACH: Registration, Evaluation, Authorization and Restriction of Chemicals; SCCS: Scientific Committee on Consumer Safety.

	EU	USA
Regulating authorities/organizations	EC; SCCS; EUON	FDA; NTF, NNI, PCPC
Relevant documents released	EC Regulation 1223/2009 [57]	Federal Food, Drug, and Cosmetic Act (FDA) [98]
	EC Recommendation 696/2011 [99]	“Considering whether an FDA-Regulated Product Involves the Application of Nanotechnology” (FDA) [100]
	SCCS Notes of Guidance 11th revision [101]	
	Guidance on the Safety Assessment of Nanomaterials in Cosmetics (SCCS) [53]	“Guidance for Industry-Safety of Nanomaterials in Cosmetic Products” (FDA) [102]
	REACH updated Regulation 1907/2006 (2018) [103]	
Nanomaterial definition	EC Regulation 1223/2009: “*an insoluble or biopersistent and intentionally manufactured material, having one or more external dimensions, or an internal structure, on the scale from 1 to 100 nm*”	No approved definition by FDA; only two points from “Considering whether an FDA-Regulated Product Involves the Application of Nanotechnology” (2014) should be used to identify nanomaterials: (1) if “*a material or final product is designed to have at least one external dimension, or internal or surface structure, in the nanoscale range (approximately 1 nm to 100 nm)*”; (2) if “*a material or final product is designed to exhibit properties or phenomena, including physical or chemical properties or biological effects, which are attributable to its size, even if these dimensions are outside the nanoscale range, down to one micrometer (1000 nm*)”.
	EC Recommendation 696/2011: *“a natural, incidental or manufactured material containing particles, in an unbound state or as an aggregate or as an agglomerate and where, for 50% or more of the particles in the number size distribution, one or more external dimensions is in the size range 1–100 nm. In specific cases and where warranted by concerns for the environment, health, safety or competitiveness the number size distribution threshold of 50% may be replaced by a threshold between 1 and 50%. By derogation from the above, fullerenes, graphene flakes and single-wall carbon nanotubes with one or more external dimensions below 1 nm should be considered as nanomaterials”*	ASTM: “*any technology that measures, manipulates or incorporates materials and/or resources from 1 to 100 nm*”
		NNI: “*nanotechnology is the development, understanding and control of materials at the nanoscale, ranging from 1 to 100 nm*”

**Table 6:** Most relevant concerns about nanomaterial safety in cosmetics. Abbreviations: D: Dermal; Exp: Intended exposure route by the applicant; I: inhalation; LO: Leave On; MBBT: Methylene Bis-Benzotriazolyl Tetramethylbutylphenol; NM: nanomaterial; O: Oral; RO: Rinse-off; SCCS: Scientific Committee on Consumer Safety; * coatings: alumina/silica, methicone/silica, aluminum hydroxide and dimethicone/methicone copolymer, trimethyloctylsilane, alumina/silicone and alumina/silica/silicone, dimethicone, simethicone, stearic acid, glycerol, dimethoxydiphenylsilane, triethoxycaprylylsilane, cetyl phosphate, manganese dioxide or triethoxycaprylylsilane.

NM	Intended Use by the Applicant	Exp	LO/ RO	Safety Concerns	SCCS Opinion	Justification	Ref
Nano-TiO_2_	UV filter in sunscreens	D/ O/ I	LO/ RO	Inhalation exposure could lead to lung inflammatory response and potential carcinogenicity	- Positive for the use up to 25% concentration - Positive for coated TiO_2_ * - Inconclusive for spray applications	Lack of dermal absorption after application to healthy, intact skin	[113,114,115]
Nano-ZnO	UV filter in sunscreens and colorant in dermally-applied products	D/ O/ I	LO/ RO	Lung inflammation after inhalation	- Positive for the use as a UV filter in sunscreens - up to 25% - concentration - Inconclusive for the use as a colorant - Negative for spray products	- No evidence for skin and oral absorption - Even if there was, continuous dissolution of zinc ions would lead to complete - solubilization of the particles in the biological environment - No information on - concentrations employed as a colorant	[116]
Nano-MBBT (120 nm)	UV filter in sunscreens, day care and whitening products	D/ O	LO/ RO	Inhalation toxicity	- Positive in concentrations - up to 10% - Caution required in case of inhalation exposure (the opinion does not apply in this case)	No concern for the dermal application with regards to the systemic effects	[117,118,128]
Tris-Biphenyl Triazine	UV filter in sunscreens and dermallyapplied products	D/ I	LO	Strong inflammatory response in the lung through inhalation exposure	- Positive up to 10% concentration, for the uncoated form with a median particle size > 80 nm - Inconclusive for spray applications	Lack of dermal absorption of the material in insoluble particulate form	[119]
Nano-Carbon black (20 nm or greater)	Colorant in skin products up to 0.001%	D/ O	LO/ RO	Toxicity, inflammation and altered phagocytosis in human monocytes	- Positive - at a concentration up to 10% - The opinion does not apply to products entailed with inhalation exposure	- General lack of skin absorption and no risk of adverse effects after application to healthy, intact skin for - nanoparticles ≥ 20 nm (the opinion applies only to such scaled nanomaterials)	[120,129]
	Colorant in nail polishes and mascaras up to 5%	D	LO				
	Colorant in other make-up eye products up to 10%	D	LO				
Nano hydroxyapatite	Toothpastes, teeth whiteners and mouthwashes up to 10%	O	RO	Cytotoxicity, induction of oxidative stress, apoptosis, and inflammatory responses	Negative/ inconclusive	- Needle-shaped nano-hydroxyapatite should not be used in cosmetics - No sufficient evidence for the other forms of nano-hydroxyapatite	[121,122,130]
Colloidal Ag	Toothpastes up to 1%	O	RO	Much of the information provided by the applicant turned out not to be relevant for safety assessment	Negative/ inconclusive	- Available information insufficient for a conclusion - Attention to the possible presence of ionic Ag in different types of finished cosmetic products	[123]
	Skin care products up to 1%	D	LO/ RO				
Nano-Silica	Absorbent & anti-caking agent, controlled release of active compounds	D/ O/ I	LO/ RO	Nanoparticles might penetrate the skin and end up in internal organs or the bloodstream—where they might be toxic	Negative/ inconclusive	No proof that nanosilica penetrates the skin or is toxic, but also no enough evidence to rule out those possibilities	[124,125]
Styrene/Acrylate Copolymer	Carrier for other bioactive compounds	D	LO	Potential accumulation of the encapsulated substances to unintended parts of the body	Negative/ inconclusive	No sufficient evidence for the safety related to nano-scale styrene/acrylates as such or as a carrier for bioactive substances	[126]
Colloidal Cu	Oral uses (mouth wash)	O	RO	Possible systemic uptake of Cu nanoparticles with potential accumulation in certain organs; potential mutagenic/genotoxic and immunotoxic/nephrotoxic effects	Negative/ inconclusive	Further safety evaluation are needed due to the relevant potential toxicological risks	[127]
